# Identification and prediction of association patterns between nutrient intake and anemia using machine learning techniques: results from a cross-sectional study with university female students from Palestine

**DOI:** 10.1007/s00394-024-03360-8

**Published:** 2024-03-21

**Authors:** Radwan Qasrawi, Manal Badrasawi, Diala Abu Al-Halawa, Stephanny Vicuna Polo, Rami Abu Khader, Haneen Al-Taweel, Reem Abu Alwafa, Rana Zahdeh, Andreas Hahn, Jan Philipp Schuchardt

**Affiliations:** 1 Department of Computer Science, Al-Quds University, Jerusalem, Palestine; 2 Department of Computer Engineering, Istinye University, Istanbul, Turkey; 3 Department of Nutrition and Food Technology, Faculty of Agriculture and Veterinary Medicine, An-Najah National University, Nablus, West Bank Palestine; 4 Department of Applied Chemistry and Biology, College of Applied Sciences, Palestine Polytechnic University, Hebron, West Bank Palestine; 5 Institute of Food Science and Human Nutrition, Leibniz University Hannover, Hannover, Germany

**Keywords:** Iron deficiency anemia, Nutrient intake, Dietary patterns, Classification and regression tree, Machine learning, K-means analysis

## Abstract

**Purpose:**

This study utilized data mining and machine learning (ML) techniques to identify new patterns and classifications of the associations between nutrient intake and anemia among university students.

**Methods:**

We employed K-means clustering analysis algorithm and Decision Tree (DT) technique to identify the association between anemia and vitamin and mineral intakes. We normalized and balanced the data based on anemia weighted clusters for improving ML models’ accuracy. In addition, t-tests and Analysis of Variance (ANOVA) were performed to identify significant differences between the clusters. We evaluated the models on a balanced dataset of 755 female participants from the Hebron district in Palestine.

**Results:**

Our study found that 34.8% of the participants were anemic. The intake of various micronutrients (i.e., folate, Vit A, B5, B6, B12, C, E, Ca, Fe, and Mg) was below RDA/AI values, which indicated an overall unbalanced malnutrition in the present cohort. Anemia was significantly associated with intakes of energy, protein, fat, Vit B1, B5, B6, C, Mg, Cu and Zn. On the other hand, intakes of protein, Vit B2, B5, B6, C, E, choline, folate, phosphorus, Mn and Zn were significantly lower in anemic than in non-anemic subjects. DT classification models for vitamins and minerals (accuracy rate: 82.1%) identified an inverse association between intakes of Vit B2, B3, B5, B6, B12, E, folate, Zn, Mg, Fe and Mn and prevalence of anemia.

**Conclusions:**

Besides the nutrients commonly known to be linked to anemia—like folate, Vit B6, C, B12, or Fe—the cluster analyses in the present cohort of young female university students have also found choline, Vit E, B2, Zn, Mg, Mn, and phosphorus as additional nutrients that might relate to the development of anemia. Further research is needed to elucidate if the intake of these nutrients might influence the risk of anemia.

## Introduction

Anemia is a public health problem affecting more than two billion people worldwide. It is particularly prevalent in low- and middle-income countries and is often linked to poverty, malnutrition, and inadequate access to healthcare [1, 2]. It is defined as a reduction in healthy red blood cells and is often diagnosed by the level of hemoglobin (Hb) in the blood [3]. The causes of anemia are multifactorial, but nutrient deficiencies play a major role. Iron deficiency anemia (IDA) is the most common type of nutritional anemia and the result of dietary iron (Fe) deficiency or low bioavailability of plant-derived Fe species from food [4, 5].

Recently, there has been increased interest in the prevalence of anemia among university students, particularly in developing countries, as they may be at higher risk for anemia due to overall poor nutrition and inadequate access to healthcare [6–8]. In addition, the risk of IDA increases in university students due to poor dietary intake of Fe-rich foods, vitamin (Vit) B12 and folate deficiency, and increased demand for Fe [9, 10]. Studies have shown that university students often have undesirable food choices, with a high intake of fast food and a low intake of fruits, vegetables, and Fe-rich foods [11, 12]. This may also be due to financial constraints or lack of time to prepare meals [3, 9]. IDA mainly affects female university students in childbearing age due to the loss of Fe during menstruation [7, 13]. Sari et al. [7] found that the duration of blood loss per menstrual cycle was one of the most important factors influencing anemia in adolescent girls according to multivariate logistic regression. In addition, several studies have shown that IDA is also significantly associated with lifestyle factors such as physical activity or smoking [3, 14]. In a study conducted by Al-alimi et al. [8], smoking was found to be negatively associated with an increased risk of IDA in university students, with smoking possibly affecting Fe absorption. Female athletes, in particular, are at increased risk of IDA due to menstrual blood loss and the resulting increased Fe requirement. Regular exercise increases the body's need for Fe to support the production of red blood cells and oxygen transport to the muscles. Female athletes involved in high-intensity training or endurance sports may require even more Fe to maintain optimal performance [15–17].

Furthermore, Fe metabolism and status are influenced by nutrient interactions and food matrix effects. Nutrient interactions involve the way different nutrients interact with each other in the body, and food matrix refers to the physical structure of foods that affect nutrient bioavailability. Vit C enhances Fe absorption, whereas calcium (Ca) and phytate are known to have inhibitory effects [9, 18, 19]. However, there may be unknown nutrient interactions and food matrix effects that affect Fe metabolism and status. Therefore, there is a need to elucidate the relationships between nutrient intake patterns and the prevalence of anemia.

Data mining techniques, including cluster and classification algorithms, have been used to identify micronutrient intake patterns among anemia risk factors for the development of targeted nutritional strategies to prevent and treat IDA in university students [20]. Machine learning (ML) techniques, such as decision tree classification modeling, have also proved helpful in identifying associated risk factors and predicting the risk of IDA in this population [21, 22]. These approaches can identify patterns of key risk factors for IDA and help develop patient-specific interventions with an accuracy rate of 70% to 87% [21, 23]. Overall, data mining and ML techniques, such as random forest and support vector machine, appear to be valuable tools for identifying and predicting IDA.

Using K-means Clustering and Regression Tree (CRT) and classification models, we aimed to identify trends in such micronutrient intake patterns associated with IDA among young healthy female subjects from a Palestinian University in the Hebron region. This study is part of a larger cross-sectional study with the overall aim of assessing the nutrient supply and health status of university students from Palestine.

## Materials and methods

### Data source

The study utilized primary data from a cross-sectional study conducted at the Palestine Polytechnic University in Hebron City in 2021. We carried out this study in accordance with the Declaration of Helsinki and the study protocol was approved by the Institutional Review Board (IRB) at the Palestine Polytechnic University (reference number KA/41/2019). We collected written consent approval from participants prior to data collection. We randomly selected the participants from the University student’s registration repository by using the matriculation numbers. The participants group included female students between the ages of 18 and 30 years. Subjects who were pregnant or breastfeeding, had chronic internal diseases (including anemia forms such as sickle cell disease or thalassemia), celiac or inflammatory bowel disease and those rejecting to participate or refusing to sign the written consent were excluded from this study.

In our cluster analysis, we initially faced data imbalances with a small sample size of 145. To ad-dress this, we employed the SMOTE (Synthetic Minority Over-sampling) technique, effectively increasing our sample size to 755 participants [24]. This aligns with the O = 2^k^ heuristic for estimating sample size, where 'k' is the number of variables, ensuring a robust subject pool for effective analysis. While some literature suggests a larger size up to 60 k or 70 k for each variable for greater statistical power, our adjusted sample size adheres to these standards, balancing between statistical robustness and practical feasibility for identifying distinct cluster [25]. The SMOTE involved creating synthetic samples by interpolating between the minority class instances. The technique created the synthetic samples by selecting pairs of neighboring minority class instances and generating new samples along the line connecting them. We used the cross-validation technique was used to evaluate the model performance and avoid overfitting and reducing model generalizations. SMOTE has been widely used in various fields, including fraud detection, medical diagnosis, and image classification [26, 27]. It has been shown to improve model performance by increasing the number of minority class samples, making it easier for the model to learn the features that distinguish the minority class from the majority class. The final dataset encompassed 755 participants. We excluded participants that refused to complete the assessment.

### Study variables

We collected the study variables using a face-to-face structured questionnaire. The questionnaire included variables related to participants’ sociodemographic data (age, sex, family income, residence, marital status, university year, and student financial support), and lifestyle data (physical activity, smoking, and sleeping habits). Physical activity was assessed using the validated International Physical Activity Questionnaire (IPAQ) in the Arabic version (7 items/short form) [28]. According to the IPAQ, three categories are distinguished: inactive, minimally active, and HEPA active (health-enhancing physical activity; a high active category). For details and cut-offs see [28].

The study conducted anthropometric measurements including weight, height, and Body Mass Index (BMI), which was categorized according to the WHO classification criteria [28]. To collect blood samples, participants fasted overnight and 15 ml of blood were collected via venipuncture from an arm vein in the morning. Platelets, red and white blood cell counts, Hb levels, mean corpuscular volume and red cell distribution width were measured using the Celltac ES MEK-7300K automated hematology analyzer (Nihon Kohden, Tokyo, Japan). We set diagnostic criteria for anemia based on the WHO classification, which used Hb and ferritin levels (non-anemic: Hb ≥ 12 g/dl, ferritin ≥ 15 μg/L; anemic: Hb < 12 g/dl, ferritin < 15 μg/L) [29].

To document food consumption, all participants had to complete three 24-h recalls, including two weekdays and one weekend day. All foods and beverages consumed had to be recorded, as well as the time, place and method of preparation. Prior to the study, participants were instructed by trained dietitians on how to complete the food recall forms. The dietitians also checked the questionnaires for completeness and plausibility and interviewed the participants if there were any discrepancies. We analyzed the nutrient intake of the 24-h recall data using the EMFID software developed by Al-Quds University and the WHO, the software includes the food composition tables of five countries (Palestine, Jordan, Lebanon, Kuwait, and Bahrain) [30]. The nutrient analysis included macronutrients and micronutrients (vitamins and minerals) intake analysis. The nutrient intakes were compared with the USDA Recommended Dietary Allowance (RDA) or the Adequate Intake (AI, if there is no RDA for the nutrient) values [31]. RDAs or AIs are commonly used in studies to assess the risk of inadequate nutrient intake [32–35], offering a conservative estimate that covers the nutrient requirements of nearly all (97–98%) healthy individuals in a population, facilitating comparisons and identification of at-risk groups [31]. The nutrient intakes were grouped into two categories: ≥ RDA/AI and < RDA/AI. Additionally, median nutrient intakes falling below the RDA/AI were subcategorized into ‘very low’ and ‘low’ groups. Those with intakes less than the median were classified as ‘very low’, while those with intakes greater than the median were classified as ‘low’.

### K-means clustering algorithm

K-means clustering is an unsupervised ML algorithm used to partition a dataset into clusters [36]. K-means works by dividing the data points into a specified number of clusters, and iteratively updating the cluster centers until convergence is reached. In this study we used the K-means clustering algorithm to identify patterns and trends in the occurrence of anemia among university students in relation to nutrient intake.

Before running the K-means algorithm, we preprocessed the data to impute missing values, scale the variables to have zero mean and unit variance, and normalize data by age. Then we ran the K-means algorithm with the optimal number of clusters, and the K-means initialization method, which the literature has shown to improve its convergence rate and result in more stable solutions. We ran the algorithm for a maximum of 100 iterations, or until convergence was reached, whichever came first.

To evaluate the fit of different clusters, we used Schwarz’s Bayesian Criterion (BIC) to determine the optimal number of clusters to use when performing K-means clustering. It is based on the idea that the true number of clusters in a dataset is unknown, and that different values for the number of clusters will result in different models of the data [37]. The BIC score for each model (i.e., each value of the number of clusters) is calculated using the following formula:

1 $$BIC = n \times log\left( \frac{SSE}{n} \right) + k \times log\left( n \right),$$

In this formula, n is the number of data points, SSE is the sum of squared errors between the data points and their closest cluster centers, and k is the number of parameters in the model (including the cluster centers and the data points).

We decided the optimal number of clusters by selecting the number of clusters that resulted in the lowest BIC score. The model with the lowest BIC score is the most parsimonious, namely the model that fits the data well.

Furthermore, we determined the quality of clusters using the Silhouette Method, which is commonly used to determine the optimal number of clusters for the K-means clustering analysis technique [38]. This method involves calculating the silhouette score for each data point, which is a measure of how similar the data point is to other data points in its own cluster compared to other clusters. To calculate the silhouette score for each data point, the average distance between the data point and all other data points in its own cluster (a) was first calculated. The average distance between the data point and all data points in the nearest neighboring cluster (b) was then calculated. The silhouette score for the data point was then calculated using the formula:

2 $$\frac{{\left( {b - a} \right)}}{{\max \left( {a,b} \right)}},$$

Thereafter we averaged the silhouette scores for all data points to determine the overall silhouette score for the cluster solution. We determined the optimal number of clusters by selecting the number of clusters that resulted in the highest average silhouette score.

To validate the results of the K-means clustering, we performed additional analyses using t-tests and Analysis of Variance (ANOVA) to identify significant differences between the clusters. Additionally, we presented visualized form of the data using scatter plots to examine the distribution of the variables within each cluster.

### Classification algorithm

Decision trees (DT) are a popular and widely used technique in the field of data mining and ML. They are a form of predictive model used to make decisions based on a set of input data [39, 40]. The DT procedure creates a tree-based classification model that classifies cases into groups or predicts values of a dependent (anemia disease) variable based on values of independent (predictor) variables. The DT is a tree-like model that represents a series of decisions and their possible consequences. It is composed of a root node, branches, and leaf nodes. The root node represents the initial decision that needs to be made, and each branch represents a possible outcome of that decision. The leaf nodes represent the final decision or prediction made by the tree.

We conducted the ML classification tree by performing the Exhaustive Chi-squared Automatic Interaction Detection (exhaustive CHAID) algorithm. It is a sophisticated, non-parametric, ML approach utilized for analyzing intricate interactions among variables. Originally, Fordon Kass developed this technique in 1980, serving as an enhancement to CHAID (Chi-squared Automatic Interaction Detector) [41].

Exhaustive CHAID functions by dissecting a dataset into distinct and exhaustive subsets, subsequently creating a DT model. It employs a chi-squared based technique to ascertain the most suitable next split at every stage and continues splitting until no statistically significant splits can be identified between the independent and dependent variables, thereby epitomizing its ‘exhaustive’ nature. Unlike other algorithm, exhaustive CHAID assesses all potential splits for each predictor variable, leading to the selection of the most significant split from all the predictors [42]. Consequently, it can uncover complex multi-tier interactions between variables and deliver substantial insights into data, making it a critical instrument for researchers and data analysts across a multitude of fields such as marketing, healthcare, and social sciences. Nonetheless, it’s worth mentioning that its exhaustive approach can be computationally demanding, particularly with extensive datasets or a large number of predictors.

In this study we used exhaustive CHAID analysis to investigate the patterns of association between nutrient intakes and anemia in a normalized and weighted sample of 755 female university students. The outcome variable was anemia status (anemic vs. non-anemic), while the predictor variables included intakes of vitamins and minerals from 24-h recalls. Two classification models were designed to examine the pattern of associations: the vitamin model and the mineral model.

In each model, the maximum tree depth was set to 5, minimum number of cases 30, and three statistical output indicators (X^2^, P-Value, % and n) for each node. The models reported accuracy rates of 87%.

There are several criteria that can be used to determine the best split at each step in the tree building process. The Gini index is one of the features’ selection methods used in DT-based ML models to determine the importance of each feature in predicting the target variable. The Gini index is a measure of the impurity of a particular split in a classification and regression tree (CRT) [43]. It is used to determine the best split at each step in the tree building process. Statisticians calculate the Gini index by comparing the proportions of different classes in a split. It is minimized when the split is pure, meaning that all the instances in a particular subset belong to the same class. The Gini index is calculated using the following formula:

3 $${\text{Gini}} = {\text{i}} - \sum \left( {\text{X}} \right)^{{2}}$$

In the formula, X is the proportion of instances in class i. If the split were pure, with all the instances belonging to the same class, the Gini index would be 0. The Gini index is just one of several criteria that can be used to determine the best split in a CRT. In this study, we used the Gini method to make predictions and decisions based on university students’ data.

## Results

### Sociodemographic descriptive analysis

The results in Table 1 show the characteristics of the study sample and the distribution of socio-demographic variables. The sample consisted of 755 female university students aged 18–24 years. The results showed that the place of residence of the participants was urban for 70.1% and non-urban for 29.9%, while 30.5%, 44.4% and 25.2% reported low, average and above average family income respectively. When asked about their lifestyle, 12.5% of participants reported smoking, 28.5% reported being physically inactive, 48.2% were moderately physically active and 23.3% were HEPA active. Approximately 94% of participants reported living in student accommodation and 17.9% reported sleeping less than 6 h per night. The results in Table 1 also show mean ± SD Hb levels by socio-demographic variables.

**Table 1:** Sociodemographic, lifestyle, and health variables of the study cohort (n = 755)

Variables	Total group n (%)	Non-Anemic subgroup n (%)	Anemic subgroup n (%)	Non-Anemic subgroup Hb (g/dl) mean ± SD	Anemic subgroup Hb (g/dl) mean ± SD	F (p-value) Hb values non-anemic vs. anemic
Age (years)						
18–19	236 (31.3)	146 (61.9)	90 (38.1)	13.1 ± 1.0	12.2 ± 1.4	8.0 (0.005)
20–21	277 (36.7)	169 (61.0)	108 (39.0)	13.4 ± 0.9	12.2 ± 1.2	
22–24	242 (32.1)	177 (73.1)	65 (26.9)	13.6 ± 0.7	11.9 ± 0.5	
Total group		492 (65.2)	263 (34.8)	13.4 ± 0.9	12.1 ± 1.2	
Living status						
With family	709 (93.9)	469 (66.1)	240 (33.9)	13.4 ± 0.8	12.2 ± 1.2	15.1 (0.001)
Students housing	46 (6.1)	23 (50.0)	23 (50.0)	12.8 ± 1.5	11.8 ± 0.6	
Family income						
Low	230 (30.5)	176 (76.5)	54 (23.5)	13.2 ± 1.0	11.6 ± 1.4	7.2 (0.007)
Moderate	335 (44.4)	200 (59.7)	135 (40.3)	13.3 ± 0.9	12.4 ± 0.9	
High	190 (25.2)	116 (61.1)	74 (38.9)	13.5 ± 0.9	12.2 ± 1.4	
Screen time						
≤ 1 h	71 (9.4)	50 (70.4)	21 (29.6)	12.3 ± 1.2	12.6 ± 0.6	1.2 (0.266)
2–3 h	241 (31.9)	145 (60.2)	96 (39.8)	13.1 ± 0.9	12.3 ± 1.3	
4–5 h	177 (23.4)	106 (59.9)	71 (40.1)	13.4 ± 0.8	12.1 ± 1.1	
≥ 6 h	266 (35.2)	191 (71.8)	75 (28.2)	13.7 ± 0.8	12.0 ± 1.4	
Place of residence						
Urban	529 (70.1)	341 (64.5)	188 (35.5)	13.4 ± 0.9	12.1 ± 1.3	0.925 (0.337)
Non-urban	226 (29.9)	151 (66.8)	75 (33.2)	13.3 ± 1.0	12.2 ± 0.9	
Physical activity						
Inactive	215 (28.5)	154 (71.6)	61 (28.4)	13.4 ± 0.8	12.4 ± 1.4	90.2 (0.001)
Moderately active	364 (48.2)	242 (66.5)	122 (33.5)	13.3 ± 1.1	12.2 ± 1.2	
HEPA active	176 (23.3)	96 (54.5)	80 (45.5)	13.3 ± 0.5	11.8 ± 1.1	
Smoking						
Yes	94 (12.5)	56 (59.6)	38 (40.4)	13.1 ± 1.0	12.0 ± 0.7	3.6 (0.057)
No	661 (87.5)	436 (66.0)	225 (34.0)	13.4 ± 0.9	12.2 ± 1.3	
Sleeping 6–8 h daily						
Yes	620 (82.1)	409 (66.0)	211 (34.0)	13.3 ± 0.9	12.1 ± 1.2	28.0 (0.001)
No	135 (17.9)	83 (61.5)	52 (38.5)	13.7 ± 0.7	12.3 ± 1.0	
BMI						
Underweight	92 (12.2)	44 (47.8)	48 (52.2)	13.2 ± 0.3	12.3 ± 0.8	4.7 (0.03)
Normal	587(77.7)	393(67.0)	194(33.0)	13.4 ± 0.9	12.1 ± 1.3	
Overweight	57(7.5)	42(73.7)	15(26.3)	13.3 ± 1.1	12.7 ± 1.1	
Obese	19(2.5)	13(68.4)	6(31.6)	13.0 ± 0.7	11.4 ± 1.1	

The study reported a prevalence of anemia of 34.8% in different age groups. The prevalence of anemia was higher in the younger age groups (38.1% in the 18–19 age group and 39.0% in the 20–21 age group) than in the older age group (26.9% in the 22–24 age group). For several other sociodemographic variables (living status, family income, place of residence, sleep duration), there were no consistent patterns for differences in anemia prevalence between categories, although in some cases the differences were significant. The higher prevalence of anemia in the group with the higher physical activity (45.5%) compared with minimal activity (33.5%) and inactivity (28.4%) was also noticeable. The prevalence of anemia was also slightly higher in smokers (40.4%) than in non-smokers (34%), although the difference showed only a trend towards significance (P = 0.057). Women with weight under the average range had the highest anemia prevalence (52.2%) compared to the women with average weight (33.0%), women with weight above the average range (26.3%) and women with obesity (31.6%).

### Overall nutrient intake

Table 2 shows the descriptive analysis of nutrient intakes from the 24-h recalls. The results indicated that individuals with anemia had lower intakes of numerous micronutrients compared to individuals without anemia. The results showed significant differences in the intake of protein, several vitamins (B1, B2, B3, B5, B6, C, as well as folate and E equivalents), and certain minerals (phosphorus, manganese, and zinc) between individuals with and without anemia. Intakes of micronutrients such as Vit A (RAE), Vit B5, Vit B6, choline, folate, Vit B12, Vit C, Vit E, calcium (Ca), magnesium (Mg), potassium (K), and iron (Fe) fall below the RDA/AI and were categorized as ‘very low’ and ‘low’.

**Table 2:** Daily nutrient intake in the total cohort (n = 755) and in anemic and non-anemic sub-groups

Nutrients	Total group Mean ± SD	< RDA/AI^1^ n (%)		≥ RDA/AI n (%)	Non-anemic subgroup Mean ± SD	Anemic subgroup Mean ± SD	F(p-value) non-anemic vs. anemic
		Very low intake^a^	Low intake^b^				
Energy and macronutrients							
Calories (Kcal)	1405 ± 360	–	509 (67.4)	246 (32.6)	1414 ± 374	1392 ± 342	0.8 (0.386)
Protein (g)	52.2 ± 14.2	–	225 (29.8)	530 (70.2)	52.3 ± 14.5	52.0 ± 13.7	**7.2 (0.008)**
Carbohydrates (g)	193 ± 54.8	–	117 (15.5)	638 (84.5)	194 ± 56.5	191 ± 52.5	0.1 (0.723)
Fat (g)	49.6 ± 15.6	–	123 (16.3)	632 (83.7)	49.9 ± 16.4	49.1 ± 14.4	0.7 (0.418)
Vitamins							
Vit A RAE^1^ (µg)	150.3 ± 247.9	484 (64.1)^a^	271 (35.9)^b^		154.7 ± 259.6	144.8 ± 234	0.06 (0.813)
Vit B1 (mg)	1.9 ± 1.5	–	361 (47.8)	394 (52.2)	1.9 ± 1.5	2.0 ± 1.5	**4.5 (0.034)**
Vit B2 (mg)	2.0 ± 2.1	–	401 (53.1)	354 (46.9)	2.2 ± 2.2	1.7 ± 1.9	**19.7 (0.001)**
Vit B3 equ.^2^ (mg)	9.6 ± 3.5	–	382 (50.6)	373 (49.4)	9.1 ± 3.6	10.5 ± 3.4	**26.3 (0.001)**
Vit B5 (mg)	4.2 ± 2.5	504 (66.8)^a^	251 (33.2)^b^	–	4.5 ± 2.5	3.9 ± 2.3	**9.6 (0.002)**
Vit B6 (mg)	1.7 ± 1.3	627 (83)^a^	128 (17)^b^	–	1.8 ± 1.4	1.6 ± 1.2	**10.8 (0.001)**
Choline^1^ (mg)	136 ± 65.0	666 (88.2)^a^	89 (11.8)^b^	–	139 ± 69.4	131 ± 58.2	1.9 (0.164)
Folate equ. (µg)	135 ± 51.3	426 (56.4)^a^	329 (43.6)^b^	–	141 ± 52.8	127 ± 48.4	**8.6 (0.003)**
Vit B12 (µg)	2.0 ± 1.5	418 (55.4)^a^	337 (44.6)^b^	–	2.0 ± 1.5	1.8 ± 1.4	2.1 (0.148)
Vit C (mg)	58.8 ± 39.7	430 (57)^a^	325 (43)^b^	–	63.8 ± 43.6	51.3 ± 31.9	**6.5 (0.011)**
Vit E equ. (mg)	1.9 ± 1.0	531 (70.3)^a^	224 (29.7)^b^	–	2.1 ± 1.2	1.7 ± 0.8	**35 (0.001)**
Minerals							
Ca (mg)	418 ± 182	443 (58.7)^a^	312 (41.3)^b^	–	433 ± 186	396 ± 175	3.8 (0.051)
Mg (mg)	148 ± 51.2	201 (26.6)^a^	554 (73.4)^b^	–	151 ± 52.6	143 ± 49.0	0.5 (0.47)
P (mg)	626 ± 202	–	372 (49.3)	383 (50.7)	634 ± 218	613 ± 177	**7.8 (0.005)**
K (mg)	1413 ± 536	431 (57.1)^a^	324 (42.9)^b^		1429 ± 522	1388 ± 561	0.2 (0.654)
Cu (mg)	2.3 ± 1.9	–	197 (26.1)	558 (73.9)	2.4 ± 2.0	2.1 ± 1.8	0.1 (0.761)
Fe (mg)	7.8 ± 2.8	251 (33.2)^a^	504 (66.8)^b^	–	8.2 ± 3.1	7.2 ± 2.5	0.9 (0.333)
Mn (mg)	1.7 ± 0.9	–	317 (42)	438 (58)	1.7 ± 1.1	1.6 ± 0.6	**19.2 (0.001)**
Zn (mg)	7.0 ± 4.2	–	308 (40.8)	447 (59.2)	7.8 ± 4.9	5.7 ± 2.4	**6.6 (0.01)**

^1^RAE: Retinol activity equivalents, ^2^Equ.: equivalents

Prevalence of subjects with nutrient intakes above or below the Recommended Daily Allowance (RDA) or Adequate Intake (AI). If the median intakes of nutrients were below the RDA, the nutrient intakes were again divided into ^a^very low intake (< median intake of the total group) and ^b^low intake (> median intake of the total group)

### K-means cluster analysis of nutrient intake

We used the K-means clustering algorithm to identify nutrient intake clusters after adjusting for age and Hb level. The results of the K-means clustering yielded two clusters with distinctive characteristics. Cluster 1 consisted of individuals with nutrient intakes < RDA/AI values, while cluster 2 consisted of individuals with nutrient intakes ≥ RDA/AI, except for Vit A (RAE), choline, Vit B2, Vit B5, Vit B6, folate, Vit B12, Vit C, Vit E equ., Mg, Fe, and manganese (Mn) for which participants' intakes were classified as “Low” and “Very low” because their intakes were entirely < RDA/AI values.

**Fig. 1:**
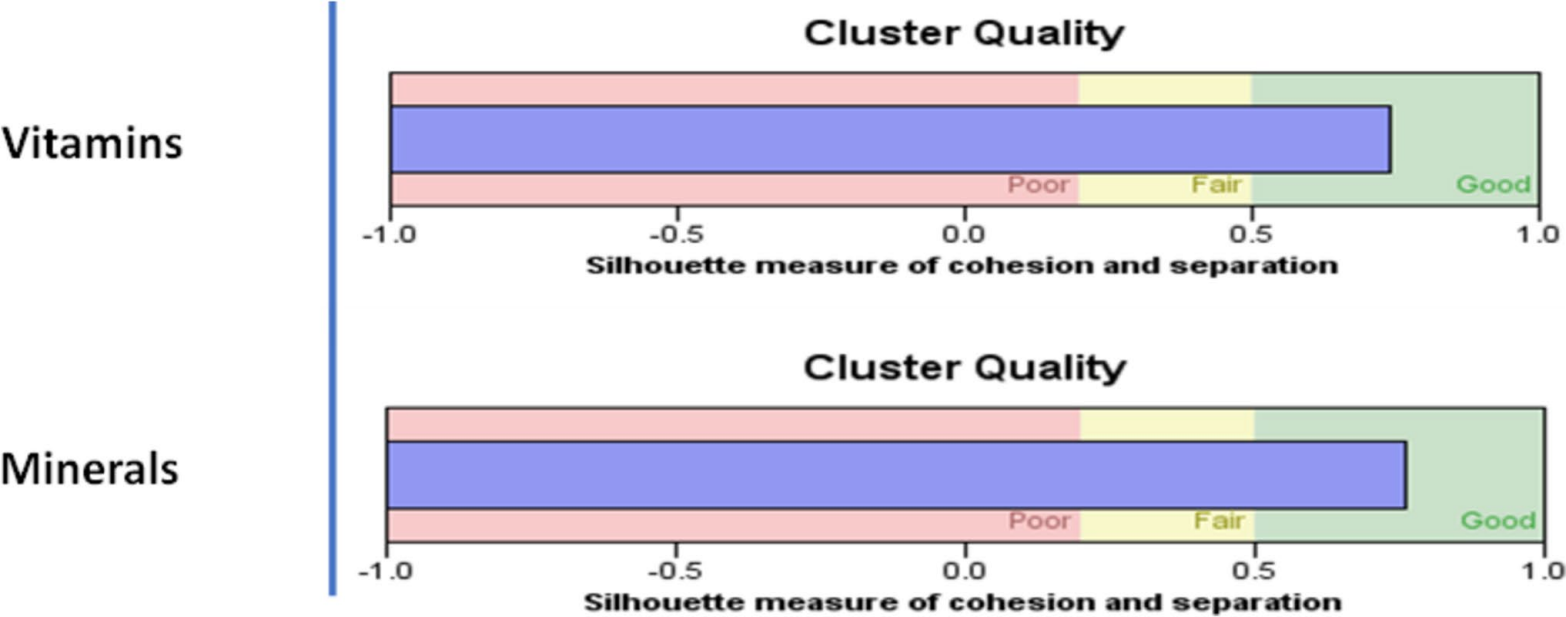
Silhouette cluster quality measures for vitamins, and minerals

We evaluated the cluster quality using the silhouette measure of cohesion and the separation algorithm. The results in Fig. 1 show the cluster quality of vitamin and mineral intake. The clustering algorithms reported high silhouette scores of 0.7 for both vitamin and mineral intake. These results show that most of the nutrients in the dataset are well matched to their own cluster, with most nutrients having a silhouette score greater than a 0.5 silhouette score, indicating that the clusters are well defined and separated.

Table 3 shows the average nutrient intakes of the students in the identified clusters. The results showed that participants in cluster 1 had significantly lower intakes of all nutrients. Multivariate analysis between the nutrient intake clusters and the variables anemia, BMI, and age revealed several associations. The analysis showed that anemia was significantly associated with energy, protein, fat, Vit B1, Vit B5, Vit B6, Vit C, Mg, copper (Cu), and zinc (Zn). BMI was significantly associated with calories, protein, carbohydrates, fat, Vit B2, Vit B3, Vit B5, Vit B6, Vit C, Mg, Cu and Zn. In addition, age was significantly associated with protein, fat, Vit B3, Vit B6, folate, Vit B12, Vit E, phosphorus (P), K, Cu, Fe, and Mn.

**Table 3:** Univariate analysis of nutrients intake clusters by anemia BMI and age

Nutrients	Cluster 1	Cluster 2	Anemia F(p-value)	BMI F(p-value)	Age F(p-value)
Energy and macronutrients					
Calories (Kcal)	1202 ± 193	1826 ± 187	**13.6 (0.001)**	**27.1 (0.001)**	0.1 (0.706)
Protein (g)	49.6 ± 10.2	78.1 ± 8.3	**4.8 (0.029)**	**7.5 (0.006)**	**13.4 (0.001)**
Carbohydrates (g)	167 ± 31.8	251 ± 29.5	3.1 (0.078)	**17.9 (0.001)**	2.3 (0.13)
Fat (g)	37.7 ± 8.1	65.2 ± 9.1	**26.9 (0.001)**	**5.4 (0.021)**	**20.7 (0.001)**
Vitamins					
Vit A RAE (µg)	45.5.4 ± 34.2^a^	170.8 ± 79.3^b^	0.5 (0.47)	0.1 (0.787)	0.2 (0.695)
Vit B1 (mg)	0.6 ± 0.2	3.2 ± 1.3	**20.9 (0.001)**	**4.9 (0.027)**	3.2 (0.073)
Vit B2 (mg)	0.8 ± 0.4	3.8 ± 1.6	0.3 (0.562)	**11.7 (0.001)**	1 (0.311)
Vit B3 equ. (mg)	9.4 ± 2.7	16.0 ± 1.9	0.5 (0.479)	**4.2 (0.041)**	**4.8 (0.029)**
Vit B5 (mg)	3.0 ± 1.0	8.1 ± 1.7	**22 (0.001)**	**36.7 (0.001)**	0.6 (0.447)
Vit B6 (mg)	1.1 ± 0.6	4.9 ± 2.7	**5 (0.026)**	**10.2 (0.001)**	**14.3 (0.001)**
Choline (mg)	82.5 ± 24.5^a^	180 ± 45.1^b^	0.6 (0.422)	**27.7 (0.001)**	0.1 (0.77)
Folate equ. (mg)	104 ± 69.4^a^	175 ± 32.8^b^	0.1 (0.79)	**6.1 (0.014)**	**6.8 (0.009)**
Vit B12 (µg)	1.2 ± 0.5^a^	5.4 ± 4.6^b^	3 (0.082)	0.9 (0.335)	**13.3 (0.001)**
Vit C (mg)	37.9 ± 17.7^a^	97.4 ± 23.5^b^	**26.4 (0.001)**	**9.5 (0.002)**	2.5 (0.113)
Vit E equ. (mg)	0.5 ± 0.5^a^	2.2 ± 0.5^b^	2.1 (0.146)	0.1 (0.703)	**14.3 (0.001)**
Minerals					
Ca (mg)	288 ± 86.6	589 ± 144	0.1 (0.812)	**17.3 (0.001)**	0 (0.856)
Mg (mg)	108 ± 22.4^a^	192 ± 39.3^b^	**14.1 (0.001)**	0.2 (0.62)	3.6 (0.059)
P (mg)	529 ± 107	882 ± 128	0.5 (0.48)	0 (0.886)	**6.1 (0.014)**
K (mg)	1,286 ± 362	2,607 ± 495	0.5 (0.466)	2.2 (0.136)	**20.2 (0.001)**
Cu (mg)	0.63 ± 0.18	2.84 ± 1.76	1.2 (0.283)	**9.9 (0.001)**	**1.7 (0.187)**
Fe (mg)	6.1 ± 1.6^a^	10.9 ± 2.8^b^	1.8 (0.181)	**9.1 (0.003)**	**7.9 (0.005)**
Mn (mg)	1.5 ± 0.8	2.6 ± 1.7	1.2 (0.279)	**17.8 (0.001)**	**14.9 (0.001)**
Zn (mg)	6.2 ± 3.1	7.5 ± 4.0	**7.1 (0.008)**	1.6 (0.207)	0 (0.866)

*Equ.* equivalents, *RAE* Retinol activity equivalents

^a^Very low intake cluster (< median intake)

^b^Low intake cluster (> median intake)

### Classification analysis of anemia and micronutrient intake

#### Anemia and vitamin model

**Fig. 2:**
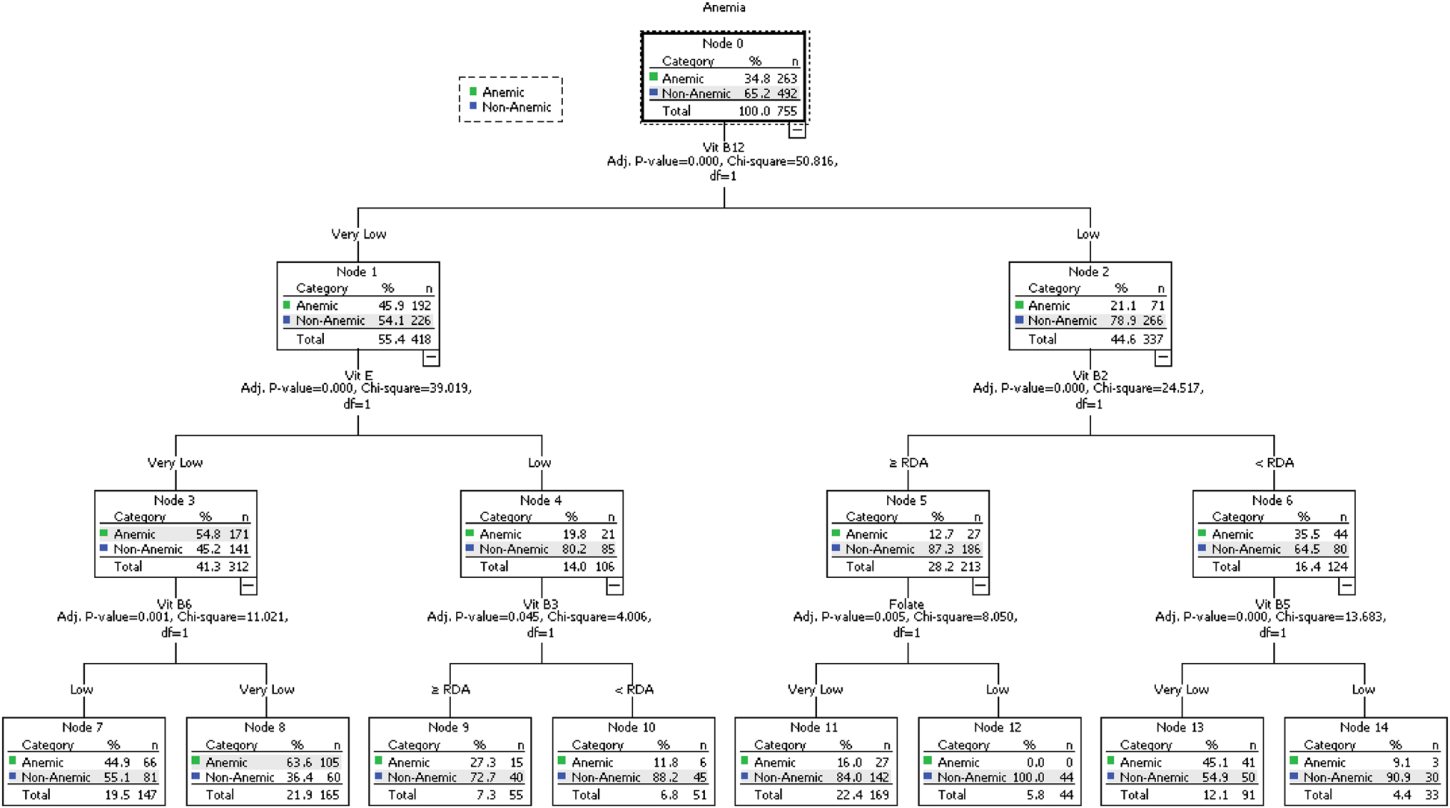
The anemia and vitamins intake decision tree classification model

Figure 2 shows the anemia classification model for vitamin intake. The classification tree identified different patterns of classification among the group of participants. The tree produced 8 terminal nodes, in which the model classified the participants according to the importance of the association between anemia and vitamin intake. The model had an accuracy rate of 82.1% with an estimated risk error of 0.322 and an SE of 0.017.

The results in Fig. 2 revealed a significant relationship between Vit B12 intake and anemia (X^2^ = 50.8, P-value < 0.001). Among the participants with a very low intake of Vit B12, the rate of anemia was higher compared to the participants with lower intake of Vit B12 (45.9% vs. 21.1%, respectively). The Vit E intake was found to be another significant factor associated with anemia (X^2^ = 39.0, P-value < 0.001). The very low Vit E intake group had a higher prevalence of anemia than the low intake group (54.8% vs. 19.8%, respectively). Vit B2 intake was also significantly associated with anemia (X^2^ = 24.5, P-value < 0.001), whereby participants with a Vit B2 intake < RDA having a higher rate of anemia than participants with a Vit B2 intake ≥ RDA (35.5% vs. 12.7%, respectively).

Interestingly, the Vit E classification groups showed significant associations with different nutrients. The very low Vit E intake group was associated with the Vit B6 intake (X^2^ = 11.0, P-value = 0.001), where the Vit B6 intake indicated that the participants with very low intakes had a higher rate of anemia compared to participants with low intake levels (63.6%vs. 44.9%, respectively). The study also found a significant association between Vit B5 intake and anemia (X^2^ = 13.7, P-value < 0.001). Of the subjects who consumed very low Vit B5, 45.1% were anemic. On the other hand, only 9.1% of those consuming low Vit B5 were anemic. Furthermore, the folate intake was also significantly associated with anemia (X^2^ = 8.1, P-value = 0.001). In the group consuming very low folate, 16.0% were anemic, whereas in the group consuming low folate, 0% were anemic.

#### Anemia and mineral model

**Fig. 3:**
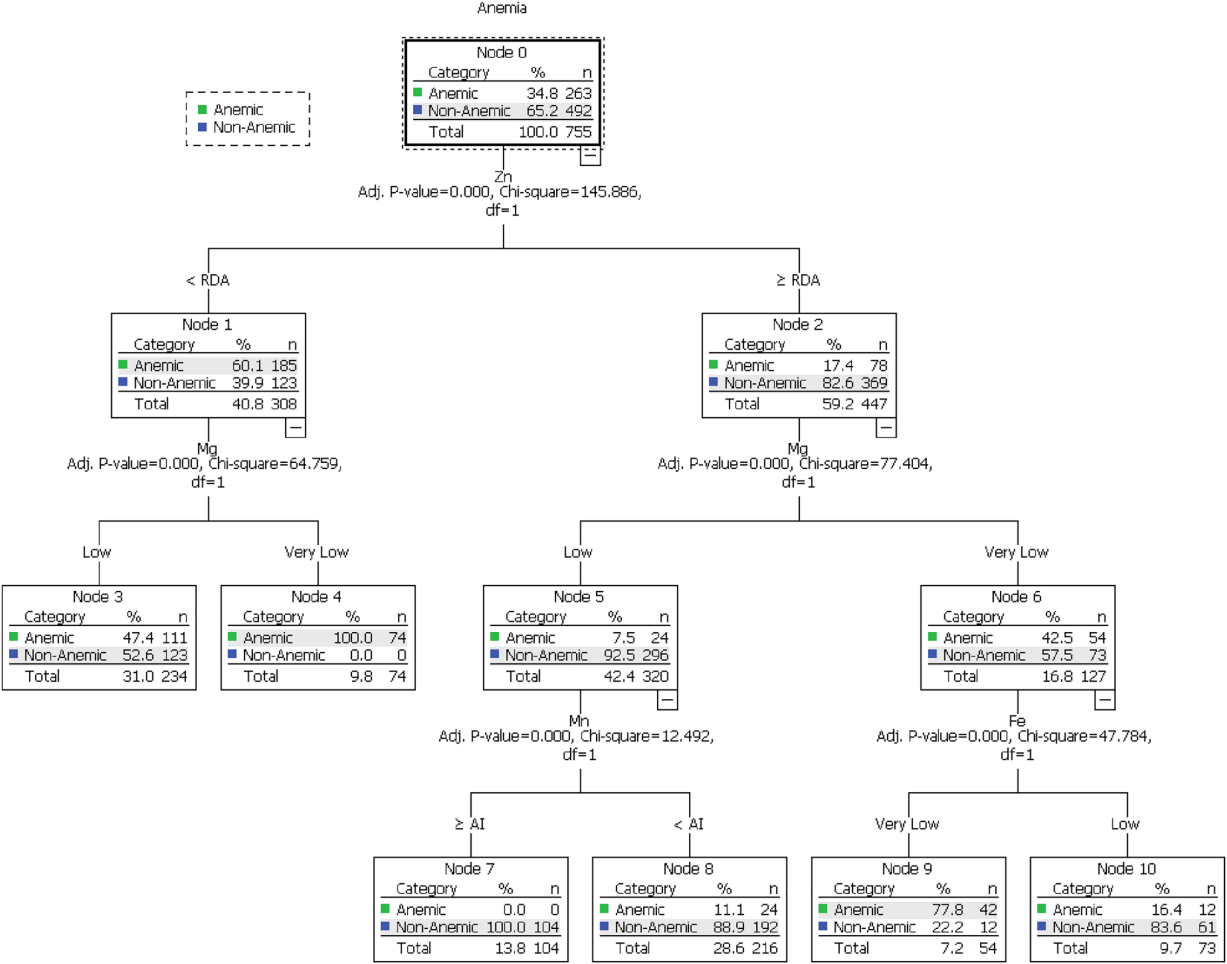
The anemia and vitamins intake decision tree classification model

Figure 3 shows the anemia classification model for mineral intake, which identified different classification patterns among participants and generated 7 terminal nodes. The model accurately classified participants based on the association between anemia and mineral intake, with an accuracy rate of 83% and an estimated risk error of 0.185 and an SE of 0.014.

The analysis in Fig. 3 revealed a significant relationship between Zn intake and anemia (X^2^ = 145.9, P-value < 0.001). Participants who consumed Zn < RDA had a higher rate of anemia than those who consumed Zn ≥ RDA (60.1% vs. 17.4%, respectively). The ≥ RDA Zn intake group was associated with the Mg intake as another significant factor associated with anemia (X^2^ = 77.4, P-value < 0.001). The very low Mg intake group had a higher rate of anemia than the low intake group (42.5% vs. 7.5%, respectively). The < RDA Zn intake group was associated with Mg intake (X^2^ = 64.8, P-value < 0.001). Participants who consumed very low Mg intake reported higher rate of anemia (100%, 47.4%, respectively).

Interestingly, the two Mg groups were significantly associated with Fe and Mn intakes. The very low Mg intake group was associated with Fe intake (X^2^ = 47.8, P-value < 0.001). Whereby the very low Fe intake group reported a higher rate of anemia than the low intake group (77.8%, 16.4%). Moreover, the Mg low intake group was associated with Mn intake (X^2^ = 12.5, P-value < 0.001). In the < AI Mn intake group, the anemia rate is higher than in the ≥ AI Mn intake group (11.1%, 0%, respectively).

### Models importance analysis of micronutrients related to anemia

**Fig. 4:**
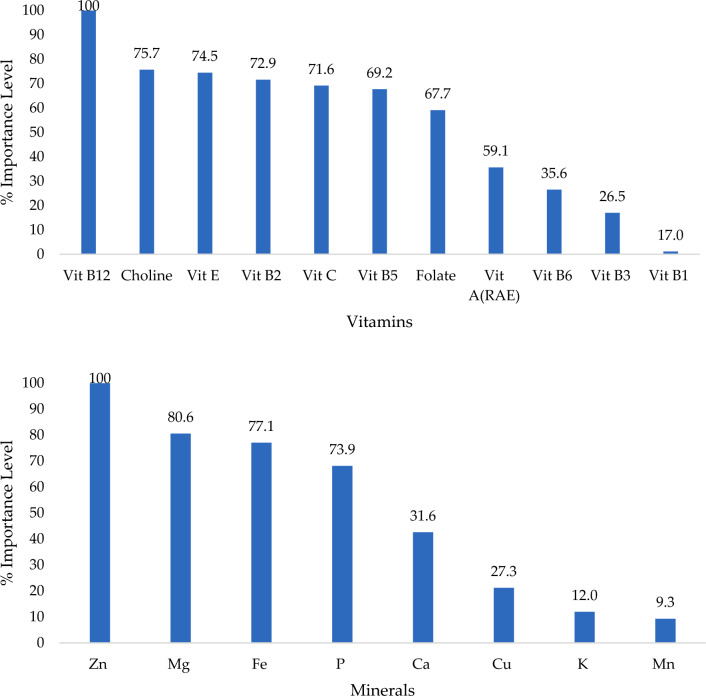
Gini Importance Analysis for (**a**) vitamins and **b** minerals

In this study we conducted the Gini Importance analysis to determine which nutrient intake factors had the greatest impact on the likelihood of developing anemia among university students (Fig. 4). The higher the score, the greater the importance of the factor in predicting anemia. The results in Fig. 4 showed the normalized importance score of the vitamin model and indicated that Vit B12, choline, Vit E, Vit B2, Vit C, Vit B5, folate, and Vit A (RAE) had a > 50% likelihood of predicting anemia. In addition, the mineral model indicated that Fe, Mg, Fe, and phosphorus (P) have a > 50% likelihood of predicting anemia.

## Discussion

The high prevalence of anemia among female university students (34.8%) is consistent with other studies investigating the prevalence of anemia, especially among female university students, in Low- and Middle-Income Countries (LMICs) [1, 44]. The literature consistently states that women, especially those of reproductive age (15–49 years), are at particularly high risk for IDA due to menstrual blood and Fe losses, pregnancy, and bleeding during childbirth [45]. Another important reason for the higher susceptibility of women in reproductive age to anemia is diet, primarily inadequate access to Fe-rich foods. In addition to Fe, other micronutrients may be associated with anemia.

In this study, we used data mining and ML techniques to identify new patterns and classifications of the associations between nutrient intake and anemia among female university students in the Hebron district in Palestine. We used the K-means clustering analysis algorithm to identify the clusters of anemia and nutrient intake. Additionally, we used the DT-ML technique to identify the classification tree between anemia and associated factors. The K-means algorithm was able to identify high quality clusters of nutrient intakes, which resulted in the identification of two main clusters (< RDA/AI; ≥ RDA/AI groups) for most of nutrients, except for those with median intakes less than the RDA/AI values, which were classified as very low and low intake clusters.

Analysis of nutrient intakes showed that median intakes of several micronutrients (including Vit A, choline, folate, Vit B12, Vit E, Vit C, Mg, Fe, an Mn) were < RDA values in the entire cohort, indicating an overall unbalanced malnutrition in the present cohort. Moreover, several nutrients were generally significantly lower in students with anemia than in students without anemia. The classification models and the Gini Importance analysis identified key nutrient factors influencing anemia, including Vit E, Vit A, Vit B12, Vit B2, Vit B3, Vit B6, Vit B5, folate, Vit C, choline, as well as Zn, Mg, Fe, Mn, and P.

The importance of micronutrients such as Fe, folate, and Vit B12 in Fe metabolism and blood cell formation is well established. Consistent with our findings, several other studies observed associations between anemia and intake of folate, Vit B12, and Fe [2, 14, 44]. An association between low intake of Vit E other than Fe, Vit B12, and folate and a higher risk of anemia in women of reproductive age and pregnant women was also described in the Women’s Health Initiative Observational Study [3].

B vitamins have important functions in the production of red blood cells and the prevention of anemia. Folate and Vit B12 play critical roles in the synthesis of DNA and red blood cells, and a deficiency of the two B vitamins can impair the production of red blood cells, leading to anemia. Vit B2 and B5 also play important roles in the Fe metabolism, albeit indirectly.

Vit B2 acts as cofactor in the conversion of vitamin B6 into its active coenzyme forms—particularly pyridoxal 5'-phosphate (PLP) and pyridoxamine 5ʹ-phosphate (PMP). Moreover, Vit B2 is involved in the conversion of the inactive form of folic acid into its active forms, such as 5-methyltetrahydrofolate (5-MTHF). Active forms of Vit B6 and folic acid are crucial for DNA synthesis, cell division, and the production of Hb and red blood cells. Vit B5 is essential for the synthesis of coenzyme A (CoA), which plays a crucial role in several metabolic pathways. Although it doesn’t directly affect Fe metabolism, a deficiency of B5 may affect overall energy production and utilization, which may indirectly affect the body's ability to maintain optimal Fe levels. Vit B6 intake was also found to be associated with the prevalence of anemia. This was particularly clear in the cluster analyses, where the prevalence of anemia was higher at very low intakes of Vit B6 than at low intakes. In addition, a comparison showed that Vit B6 intake was significantly lower in the anemic group than in the non-anemic group. However, in the Gini importance analysis, Vit B6 intake in this cohort was less important than expected. There is no physiological reason for this unexpected finding. The fact that the intake of Vit B6 was extremely low in the whole cohort (median intake < RDA) is probably more responsible. 88% of the subjects had a Vit B6 intake that could be classified as very low. Compared with the other nutrients, intakes of Vit B6 were the worst.

The Vit C intake of the entire cohort was low (median intake < RDA). In addition, Vit C intake was significantly lower in the anemic group than in the non-anemic group. Vit C intake was significantly associated with anemia across the cohort, although the Vit C node doesn’t appear in the tree model due to the small sample of participants. Vit C plays a significant role in Fe metabolism and absorption. It significantly improves the bioavailability of dietary Fe, especially plant-derived non-heme Fe by converting ferric Fe (less absorbable form) into ferrous Fe (more absorbable form) [46]. Therefore, the low intake of Vit C in the present cohort is likely to contribute to the high prevalence of anemia.

Choline is a vitamin-like nutrient found in many foods, however, because choline can also be formed in the liver, no RDA values have been published. The USDA specifies an AI of 425 mg/day for choline for women aged 19 years and older [47]. In the USA, the average daily intake of choline from food and beverages is 278 mg for women. At 136 mg, the daily choline intake in the present cohort of female Palestinian students is only half of this and only one third of the AI. Our data show that choline intake is associated with the rate of anemia. As with Vit C, choline does not appear as a node in the tree model due to the overall small number of cases. Choline is involved in three major physiological processes: structural integrity and lipid-derived signaling for cell membranes, cholinergic neurotransmission, and methylation [48]. Choline deficiency can lead to muscle damage, liver damage, and nonalcoholic fatty liver disease. The exact physiological relationships between choline metabolism and the development of anemia are unclear. A possible link has been discussed based on the involvement of choline in the de novo metabolism of pyrimidines. Pyrimidine deficiency reduces the stability of red blood cells, which is a hallmark of anemia [48]. Studies in rats have shown that choline supplementation reduces the effects of iron deficiency [49]. The role of choline for anemia in adults remains unclear and should be investigated in future studies.

We also found associations between anemia and the intake of the two fat-soluble vitamins, Vit E and Vit A. It has observed that mild to moderate Vit E deficiency is common in women of reproductive age in a population in South Asia [50]. The role of Vit E in the prevention and treatment of anemia has not been clearly established. There is a debate whether Vit E acts as an antioxidant in red blood cell membranes, preventing the oxidation of polyunsaturated fatty acids, and thereby inhibiting premature erythrocyte lysis [51]. Healthy red blood cells are essential for maintaining proper iron levels and preventing certain types of anemia. Clinical studies have shown that Vit E acts as an erythropoietic agent, reducing the fragility of red blood cells [52, 53]. Vit A also appears to play an important role in the pathogenesis of anemia by increasing the mobilization of Fe stores and the growth and differentiation of red blood cell precursors [54]. In addition, Vit A enhances immunity to infection, thereby reducing susceptibility to infectious anemia. Epidemiologic studies have shown that the prevalence of anemia in populations in developing countries is increased by Vit A deficiency [54]. Daily intakes of both vitamins are well below the RDAs in the overall cohort and especially in women with anemia. The clinical status of both vitamins was not assessed, but because of the extremely low intakes, deficiencies of both vitamins can be expected.

Our classification mineral intake model showed that low (or very low) intakes of Zn, Mg, Fe, and Mn were associated with a higher risk of anemia, while the Gini Importance analysis revealed that the most important factors in predicting anemia were Zn, Mg, Fe, and P. The Fe intakes in the low intake cluster (6.1 ± 1.6 mg/d) and in the anemia group (7.2 ± 2.5 mg/d) were well below the RDA values for Fe for menstruating women (15–18 mg/d). The available data cannot be used to clarify the contribution of poorly bioavailable plant-derived Fe or more readily available heme-bound Fe to the total intake. In any case, our results confirm that an inadequate consumption of Fe-rich foods is associated with the prevalence of anemia in university students, which has been determined in comparable studies [11, 12].

With respect to Fe, Zn, and Mg, our findings are consistent with other studies that have found a significant association between anemia and an inadequate intake or deficiency of these minerals [14, 18, 46–48]. It is known from experimental studies that high doses of minerals such as Ca, Zn, Cu, or Mn can inhibit the absorption of non-heme (plant-derived) Fe. In a normal, balanced diet, these effects are insignificant. In the present cohort contrast, however, dietary intakes of several of these minerals (e.g., Mg, Fe) are well below the RDA values. Our finding that the prevalence of anemia is higher in the very low intake clusters of the corresponding minerals than in the low intake clusters is therefore plausible. A physiological explanation could be due to the essential functions of the various minerals in Fe metabolism and red blood cell formation. For example, the trace element Zn plays an important role in Fe metabolism and the prevention of anemia in several ways. Zn is involved in the regulation of Fe absorption in the intestine and is crucial for the storage and mobilization of Fe in the body [55, 56]. For example, it interacts with the Fe-storing protein ferritin and with hepcidin, which regulates Fe absorption and release. Zn is also a co-factor for enzymes involved in heme synthesis and Fe metabolism. Several studies, including animal models, suggest that Zn is essential for erythropoiesis [57]. In most cases, Fe deficiency coexists with Zn deficiency and there is evidence that Zn deficiency is a major contributor to Fe deficiency anemia [56]. Therefore, a combination of Fe and Zn supplementation, rather than Fe replacement alone, may be considered for more effective treatment of IDA [58].

Mg is a cofactor for various enzymes involved in numerous metabolic pathways and is therefore involved in the regulation of cell replication, differentiation, and apoptosis [59] and the hematopoietic system [59]. A Mg imbalance or deficiency can lead to modification of increased oxidative stress [60] and inflammation [61], which in turn is associated with anemia. Cross-sectional studies have shown that a high intake of Mg is negatively associated with the presence of anemia [62], suggesting that Mg may play an important role in the development of anemia. Finally, the Gini Importance analysis identified P as a nutrient whose intake is also considered a predictor of anemia. High P intakes may influence the bioavailability of Fe [63]. However, the intake of P for the entire cohort was 626 ± 202 mg/d, which is well below the RDA of 1250 mg/d. Therefore, an absorption-inhibiting influence of P on Fe is unlikely. Similarly, the differences in mean P intakes between the non-anemia and anemia groups are marginal. A link between P intake and IDA is rather unlikely.

## Conclusions

The prevalence of anemia of among female university students from Hebron district in Palestine was very high at 34.8%. The dietary quality of the entire cohort was poor. Women with anemia had an unbalanced diet with many micronutrients below the RDA/AI. In addition to nutrients known to be associated with anemia, such as folate, Vit B6, Vit C, Fe, and Vit B12, our cluster analyses also identified choline, Vit E, Vit B2, Zn, Mg, Mn, and P as other nutrients whose intake may also be associated with the occurrence of anemia. Ultimately, this question cannot be answered here, as markers of nutrient status would need to be collected to clarify these relationships. Future studies should, for example, clarify the connection between low choline intake and the risk of anemia. Our study highlights the potential of data mining and ML techniques to identify patterns and classifications of the associations between nutrient intake and anemia.

## Study limitations

Our study has several limitations, including the use of cross-sectional data, which provides only a snapshot of the participants’ nutritional status and anemia prevalence at one time point. As the ML algorithms take all nutrients into account, associations between nutrient intakes and anemia prevalence may be identified that have no known physiological relationship. Interpretation of the results and combination with other research methods is therefore crucial to draw accurate conclusions. Moreover, the study did not examine potential confounding variables that could have influenced the study results. The study relied on self-reported dietary intake data, which may be subject to recall bias and misreporting. Additionally, the sample size of the study was relatively small, which limits the generalizability of the findings to other university students in Palestine in general. Finally, the study did not control other potential factors that may influence anemia risk, such as genetic predisposition or medication use.

## Data Availability

The datasets in the present study can be obtained from the corresponding author upon a reasonable request.

## References

[1] WHO et al (2011) Prevalence of iron deficiency and i3ron deficiency anemia among females at university stage. J Pak Med Assoc 4(1):2005–2006. 10.5005/jp-journals-10006-1177 10.5005/jp-journals-10006-1177

[2] Bhadra P, Deb A (2020) A review on nutritional anemia. Indian J Nat Sci 10(59):18675–18681

[3] Thomson CA et al (2011) Nutrient intake and anemia risk in the women’s health initiative observational study. J Am Diet Assoc 111(4):532–541. 10.1016/j.jada.2011.01.017 21443985 10.1016/j.jada.2011.01.017 PMC3066454

[4] Al Hassand N (2015) The prevalence of iron deficiency anemia in a Saudi University female students. J Microsc Ultrastruct 3(1):25. 10.1016/j.jmau.2014.11.003 30023178 10.1016/j.jmau.2014.11.003 PMC6014218

[5] Cembranel F, Corso ACT, González-Chica DA (2017) Inadequacies in the treatment of iron deficiency anemia among children registered in the national program of iron supplementation in Florianopolis, Santa Catarina, Brazil. Texto e Contexto Enfermagem 26(2):1–11. 10.1590/0104-07072017006310015 10.1590/0104-07072017006310015

[6] Hamali HA et al (2020) Prevalence of anemia among Jazan university students. Int J Gen Med 13:765–770. 10.2147/IJGM.S275702 33116767 10.2147/IJGM.S275702 PMC7547133

[7] Sari P, Herawati DMD, Dhamayanti M, Hilmanto D (2022) Anemia among Adolescent Girls in West Java, Indonesia: Related Factors and Consequences on the Quality of Life. Nutrients 14(18):1–13. 10.3390/nu14183777 10.3390/nu14183777 PMC9503484 36145153

[8] Al-Alimi AA, Bashanfer S, Morish MA (2018) Prevalence of iron deficiency anemia among university students in Hodeida Province, Yemen. Anemia. 10.1155/2018/4157876 29850236 10.1155/2018/4157876 PMC5937585

[9] Hoey L et al (2007) Effect of a voluntary food fortification policy on folate, related B vitamin status, and homocysteine in healthy adults. Am J Clin Nutr 86(5):1405–1413. 10.1093/ajcn/86.5.1405 17991653 10.1093/ajcn/86.5.1405

[10] Swaminathan S, Ghosh S, Varghese JS, Sachdev HS, Kurpad AV, Thomas T (2019) Dietary iron intake and anemia are weakly associated, limiting effective iron fortification strategies in India. J Nutr 149(5):831–839. 10.1093/jn/nxz009 31050752 10.1093/jn/nxz009

[11] Shill KB et al (2014) Prevalence of iron-deficiency anaemia among university students in Noakhali Region, Bangladesh. J Health Popul Nutr 32(1):103–110 24847599 PMC4089078

[12] Hwalla N et al (2017) The prevalence of micronutrient deficiencies and inadequacies in the middle east and approaches to interventions. Nutrients 9(3):1–28. 10.3390/nu9030229 10.3390/nu9030229

[13] Meena K, Tayal DK, Gupta V, Fatima A (2019) Using classification techniques for statistical analysis of Anemia. Artif Intell Med 94:138–152. 10.1016/j.artmed.2019.02.005 30871679 10.1016/j.artmed.2019.02.005

[14] Qasrawi R, Abu Al-Halawa D (2022) Cluster analysis and classification model of nutritional anemia associated risk factors among Palestinian schoolchildren, 2014. Front Nutr 9:1–11. 10.3389/fnut.2022.838937 10.3389/fnut.2022.838937 PMC9127973 35619964

[15] Saarela M, Jauhiainen S (2021) Comparison of feature importance measures as explanations for classification models. SN Appl Sci 3(2):1–12. 10.1007/s42452-021-04148-9 10.1007/s42452-021-04148-9

[16] Pal S, Rishi P, Pawaria S, Das J, Relayach N (2020) Prevalence of iron deficiency with or without anemia in female athletes—a review. Eur J Mol Clin Med 7(11):2586–2595

[17] Nicotra D, Arieli R, Redlich N, Navot-Mintzer D, Constantini NW (2023) Iron deficiency and anemia in male and female adolescent athletes who engage in ball games. J Clin Med 12(3):4–11. 10.3390/jcm12030970 10.3390/jcm12030970 PMC9918288 36769618

[18] Houghton LA, Parnell WR, Thomson CD, Green TJ, Gibson RS (2016) Serum zinc is a major predictor of anemia and mediates the effect of selenium on hemoglobin in school-aged children in a nationally representative survey in New Zealand. J Nutr 146(9):1670–1676. 10.3945/jn.116.235127 27466609 10.3945/jn.116.235127

[19] Nasreddine LM, Kassis AN, Ayoub JJ, Naja FA, Hwalla NC (2018) Nutritional status and dietary intakes of children amid the nutrition transition: the case of the Eastern Mediterranean Region. Nutr Res 57:12–27. 10.1016/j.nutres.2018.04.016 30122192 10.1016/j.nutres.2018.04.016

[20] Sasikala N, Banu GR, Babiker T, Rajpoot P (2021) A role of data mining techniques to predict anemia disease. Int J Comput Appl 174(20):16–20. 10.5120/ijca2021921090 10.5120/ijca2021921090

[21] Rahman Khan J, Chowdhury S, Islam H, Raheem E (2022) Machine learning algorithms to predict the childhood anemia in Bangladesh. J Data Sci 17(1):195–218. 10.6339/JDS.201901_17(1).0009 10.6339/JDS.201901_17(1).0009

[22] Karagül Yıldız T, Yurtay N, Öneç B (2021) Classifying anemia types using artificial learning methods. Eng Sci Technol Int J 24(1):50–70. 10.1016/j.jestch.2020.12.003 10.1016/j.jestch.2020.12.003

[23] Yu CH, Bhatnagar M, Hogen R, Mao D, Farzindar A, Dhanireddy K (2018) Anemic status prediction using multilayer perceptron neural network model. GCAI 50:204–213. 10.29007/8bh6 10.29007/8bh6

[24] Chawla NV, Bowyer KW, Hall LO, Kegelmeyer WP (2011) SMOTE: synthetic minority over-sampling technique. J Artif Intell Res 16:321–357. 10.1613/jair.953 10.1613/jair.953

[25] Dolnicar S, Grün B, Leisch F, Schmidt K (2014) Required sample sizes for data-driven market segmentation analyses in tourism. J Travel Res 53(3):296–306. 10.1177/0047287513496475 10.1177/0047287513496475

[26] Gao C, Fei CJ, McCarl BA, Leatham DJ (2020) Identifying vulnerable households using machine-learning. Sustainability (Switzerland) 12(15):1–18. 10.3390/su12156002 10.3390/su12156002

[27] Kebede Kassaw A, Yimer A, Abey W, Molla TL, Zemariam AB (2023) The application of machine learning approaches to determine the predictors of anemia among under five children in Ethiopia. Sci Rep 13(1):1–10. 10.1038/s41598-023-50128-x 38129535 10.1038/s41598-023-50128-x PMC10739802

[28] Helou K, El El Helou N, Mahfouz M, Mahfouz Y, Salameh P, Harmouche-Karaki M (2017) Validity and reliability of an adapted Arabic version of the long international physical activity questionnaire. BMC Public Health. 10.1186/s12889-017-4599-7 28738790 10.1186/s12889-017-4599-7 PMC5525276

[29] WHO. Archived: iron deficiency anaemia: assessment, prevention and control. https://www.who.int/publications/m/item/iron-children-6to23--archived-iron-deficiency-anaemia-assessment-prevention-and-control. Accessed 25 Feb 2024

[30] Alquds University and WHO. Eastern Mediterranean Food Information Databank (EMFID). https://emfid.org/frontend/web/index.php?r=site/index. Accessed 25 Feb 2024

[31] National Institutes of Health, Office of Dietary Supplements. (n.d.). Nutrient Recommendations: Dietary Reference Intakes (DRI) and Recommended Dietary Allowances (RDA). https://ods.od.nih.gov/HealthInformation/nutrientrecommendations.aspx#databases. Accessed 20 Jan 2024

[32] Al Masri F, Müller M, Straka D, Hahn A, Schuchardt JP (2022) Nutritional and health status of adult Syrian refugees in the early years of asylum in Germany: a cross-sectional pilot study. BMC Public Health 22(1):1–15. 10.1186/S12889-022-14684-7/TABLES/5 36447164 10.1186/S12889-022-14684-7/TABLES/5 PMC9706931

[33] Bruns A, Nebl J, Jonas W, Hahn A, Schuchardt JP (2023) Nutritional status of flexitarians compared to vegans and omnivores—a cross-sectional pilot study. BMC Nutr 9(1):1–14. 10.1186/S40795-023-00799-6/TABLES/5 38017527 10.1186/S40795-023-00799-6/TABLES/5 PMC10685640

[34] Shankar H et al (2019) Association of dietary intake below recommendations and micronutrient deficiencies during pregnancy and low birthweight. J Perinat Med 47(7):724–731. 10.1515/JPM-2019-0053/MACHINEREADABLECITATION/RIS 31318696 10.1515/JPM-2019-0053/MACHINEREADABLECITATION/RIS

[35] Gupta A, Noronha JA, Shobha, Garg M (2018) Dietary intake of macronutrients and micronutrients among adolescent girls: a cross sectional study. Clin Epidemiol Glob Health 6(4):192–197. 10.1016/J.CEGH.2018.02.010 10.1016/J.CEGH.2018.02.010

[36] Sinaga KP, Yang MS (2020) Unsupervised K-means clustering algorithm. IEEE Access 8:80716–80727. 10.1109/ACCESS.2020.2988796 10.1109/ACCESS.2020.2988796

[37] Neath AA, Cavanaugh JE (2012) The Bayesian information criterion: background, derivation, and applications. Wiley Interdiscip Rev Comput Stat 4(2):199–203. 10.1002/wics.199 10.1002/wics.199

[38] Thinsungnoen T, Kaoungku N, Durongdumronchai P, Kerdprasop K, Kerdprasop N (2015) The clustering validity with silhouette and sum of squared errors. pp 44–51. 10.12792/iciae2015.012.

[39] Charbuty B, Abdulazeez A (2021) Classification based on decision tree algorithm for machine learning. J Appl Sci Technol Trends 2(01):20–28. 10.38094/jastt20165 10.38094/jastt20165

[40] DeGregory KW et al (2018) A review of machine learning in obesity. Obes Rev 19(5):668–685. 10.1111/obr.12667 29426065 10.1111/obr.12667 PMC8176949

[41] Kass GV. An exploratory technique for investigating large quantities of categorical data. Kass GV. Published by : Wiley for the Royal Statistical Society Stable. http://www.jstor.org/stable/2986296 An exploratory technique for investigating L. Journal of the Roral Statistical Society, vol. 29, no. 2, pp. 119–127, 1980.

[42] Biggs D, De Ville B, Suen E (1991) A method of choosing multiway partitions for classification and decision trees. J Appl Stat 18(1):49–62. 10.1080/02664769100000005 10.1080/02664769100000005

[43] Nembrini S, König IR, Wright MN (2018) The revival of the Gini importance? Bioinformatics 34(21):3711–3718. 10.1093/BIOINFORMATICS/BTY373 29757357 10.1093/BIOINFORMATICS/BTY373 PMC6198850

[44] Shams S et al (2010) The prevalence of iron deficiency anaemia in female medical students in Tehran. Singapore Med J 51(2):116–119 20358149

[45] WHO. Nutritional anaemias: tools for effective prevention and control nutritional anaemias: tools for effective prevention and control. https://www.who.int/publications/i/item/9789241513067. Accessed 25 Feb 2024.

[46] Skolmowska D, Głąbska D (2022) Effectiveness of dietary intervention with iron and vitamin C administered separately in improving iron status in young women. Int J Environ Res Public Health 19(19):1–19. 10.3390/ijerph191911877 10.3390/ijerph191911877 PMC9564482 36231177

[47] O. B. V. and Choline. Institute of Medicine (US) Standing Committee on the Scientific Evaluation of Dietary Reference Intakes and its Panel on Folate. Dietary Reference Intakes for Thiamin, Riboflavin, Niacin, Vitamin B6, Folate, Vitamin B12, Pantothenic Acid, Biotin, and Choline. National Academy Press Washington, DC; 1998. 10.17226/6015. 23193625

[48] Wortmann SB, Mayr JA (2019) Choline-related-inherited metabolic diseases A mini review. J Inherit Metab Dis 42(2):237–242. 10.1002/jimd.12011 30681159 10.1002/jimd.12011 PMC7814885

[49] Tran PV et al (2016) Prenatal choline supplementation diminishes early-life iron deficiency-induced reprogramming of molecular networks associated with behavioral abnormalities in the adult rat hippocampus. J Nutr 146(3):484–493. 10.3945/jn.115.227561 26865644 10.3945/jn.115.227561 PMC4763487

[50] Jilani T, Iqbal MP (2018) Vitamin E deficiency in south asian population and the therapeutic use of alpha-tocopherol (Vitamin E) for correction of anemia. Pak J Med Sci 34(6):1571–1575. 10.12669/pjms.346.15880 30559825 10.12669/pjms.346.15880 PMC6290196

[51] Collins AE, Saleh TM, Kalisch BE (2022) Naturally occurring antioxidant therapy in Alzheimer’s disease. Antioxidants. 10.3390/antiox11020213 35204096 10.3390/antiox11020213 PMC8868221

[52] Toprak O (2006) Effect of vitamin E therapy on oxidative stress and erythrocyte osmotic fragility in patients on peritoneal dialysis and hemodialysis. https://www.researchgate.net/publication/6627320 17173246

[53] Iqbal TP (2011) Does vitamin E have a role in treatment and prevention of anemia? http://ecommons.aku.edu/pakistan_fhs_mc_bbs 21454177

[54] Semba RD, Bloem MW (2002) The anemia of vitamin a deficiency: epidemiology and pathogenesis. Eur J Clin Nutr 56(4):271–281. 10.1038/sj.ejcn.1601320 11965502 10.1038/sj.ejcn.1601320

[55] Kondaiah P, Yaduvanshi PS, Sharp PA, Pullakhandam R (2019) Iron and zinc homeostasis and interactions: does enteric zinc excretion cross-talk with intestinal iron absorption? Nutrients. 10.3390/nu11081885 31412634 10.3390/nu11081885 PMC6722515

[56] Knez M, Graham RD, Welch RM, Stangoulis JCR (2017) New perspectives on the regulation of iron absorption via cellular zinc concentrations in humans. Crit Rev Food Sci Nutr 57(10):2128–2143. 10.1080/10408398.2015.1050483 26177050 10.1080/10408398.2015.1050483

[57] Jeng SS, Chen YH (2022) Association of Zinc with Anemia. Nutrients 14(22):1–18. 10.3390/nu14224918 10.3390/nu14224918 PMC9696717 36432604

[58] Abdelhaleim AF, Amer AF, Abdo Soliman JS (2019) Association of zinc deficiency with iron deficiency anemia and its symptoms: results from a case-control study. Cureus 11(1):1–5. 10.7759/cureus.3811 10.7759/cureus.3811 PMC6402732 30868025

[59] da Silva Lima F et al (2018) An insight into the role of magnesium in the immunomodulatory properties of mesenchymal stem cells. J Nutr Biochem 55:200–208. 10.1016/j.jnutbio.2018.02.006 29554498 10.1016/j.jnutbio.2018.02.006

[60] Zheltova AA, Kharitonova MV, Iezhitsa IN, Spasov AA (2016) Magnesium deficiency and oxidative stress: an update. BioMedicine (Taiwan) 6(4):8–14. 10.7603/s40681-016-0020-6 10.7603/s40681-016-0020-6 PMC5112180 27854048

[61] King DE, Mainous AG, Geesey ME, Ellis T (2007) Magnesium intake and serum C-reactive protein levels in children. Magnes Res 20(1):32–36. 10.1684/mrh.2007.0090 17536486 10.1684/mrh.2007.0090

[62] Shi Z, Hu X, He K, Yuan B, Garg M (2008) Joint association of magnesium and iron intake with anemia among Chinese adults. Nutrition 24(10):977–984. 10.1016/j.nut.2008.05.002 18586459 10.1016/j.nut.2008.05.002

[63] Bour NJS, Soullier BA, Zemel MB (1984) Effect of level and form of phosphorus and level of calcium intake on zinc, iron and copper bioavailability in man. Nutr Res 4(3):371–379. 10.1016/S0271-5317(84)80098-6 10.1016/S0271-5317(84)80098-6

